# Feedback-based training reduces ensemble perception bias of facial emotions in individuals with high social anxiety: A single-session randomized controlled trial

**DOI:** 10.1371/journal.pone.0337108

**Published:** 2025-12-03

**Authors:** Jisu Choi, Jae-Won Yang

**Affiliations:** Department of Psychology, The Catholic University of Korea, Bucheon-si, Wonmi-gu, Gyeonggi-do, Republic of Korea; The Chinese University of Hong Kong, HONG KONG

## Abstract

People can efficiently extract summary statistics from a set of objects—a process known as ensemble perception—including the average emotion of a facial crowd. Individuals with high social anxiety, however, tend to perceive facial crowds as more negative, reflecting a systematic perceptual bias. Because the ability to interpret group emotions is important to adaptive social functioning, this study examined whether a feedback-based training paradigm could reduce ensemble perception bias, and whether its effects varied by social anxiety. A total of 120 Korean university students were randomly assigned to either a training (*n* = 60) or control (*n* = 60) condition. Participants first completed baseline questionnaires assessing trait and state social anxiety and depression, then performed an ensemble perception task. In the training condition, participants rated the mean emotional intensity of a facial crowd on a continuous scale and received visual feedback displaying both their rating and the actual mean intensity. The control group performed the same task without feedback. Ensemble perception bias and absolute error were assessed before and after training. State social anxiety was reassessed after the task. Overall, feedback training significantly reduced bias and marginally reduced error but did not affect state social anxiety. Although formal moderation by trait social anxiety as a continuous variable was non-significant, exploratory subgroup analyses revealed that participants with high social anxiety showed notable reductions in bias, whereas no such changes were observed in the low social anxiety group. These effects remained after controlling for depressive symptoms. These findings suggest that individuals with high social anxiety may be particularly responsive to corrective feedback, enabling recalibration of their perceptual tendencies. Accordingly, feedback-based training may represent a promising approach for reducing perceptual bias in social perception among socially anxious individuals.

## Introduction

We are constantly exposed to a vast amount of visual information. However, our visual system has limited processing capacity, preventing us from perceiving every detail in a scene. Instead, we extract summary statistics from a set of objects—a process known as ensemble perception or ensemble coding [1]. Research shows that ensemble perception occurs across various levels of visual features. For example, individuals can rapidly perceive the average direction of motion in a group of moving objects [2] as well as higher-level features, such as the overall emotional tone of a crowd of faces, which is highly relevant to social life [3,4]. Previous studies suggest that individual differences—such as gender, cultural background, and psychological state—influence ensemble perception of facial crowds [5–7], and this process is thought to be important for adapting to social environments [8,9].

In this study, we focus on social anxiety, which is characterized by an intense fear of social situations where one may be scrutinized by others [10]. Individuals with social anxiety tend to focus on external cues suggesting negative evaluation when perceiving an imagined or actual audience in social settings [11]. Research has shown that socially anxious individuals perceive crowds of negative faces as more negative [12] and extract the average emotion of facial crowds as more negative than those with low social anxiety [13,14]. Such negative perceptual distortion could lead to maladaptive social responses (e.g., avoidance or withdrawal) [15]. These findings collectively suggest that ensemble perception may serve as a key cognitive mechanism linking biased social perception to social anxiety.

Given this relationship, correcting distorted ensemble perception may offer a promising avenue for intervention. However, to date, few studies have explored intervention methods targeting ensemble perception of facial emotions. The present study introduces a feedback-based training paradigm as a novel approach, extending prior work that focused on single-face emotion perception [16,17]. In this paradigm, emotional intensity was manipulated by morphing facial expressions, incrementally changing from 100% negative to 100% positive emotion. Instead of using forced-choice categorization (e.g., labeling an ambiguous face as either “happy” or “sad”), participants rated their perceived mean emotional intensity of a facial crowd on a continuous scale. The training provided individualized corrective feedback that allowed them to visually and directly compare their rating with the actual objective intensity of the stimulus on the same scale. This method goes beyond simple categorical feedback (i.e., “correct/incorrect”) by providing information on both the magnitude and direction of the error. It was found that this feedback training could correct misperception of a single face in socially anxious individuals [16].

To clarify the target of this intervention, a conceptual distinction must be made between error and bias. Error can be either non-systematic (random noise) or systematic (a consistent deviation, i.e., bias). While non-systematic errors may be difficult to rectify in the short term, a systematic bias can be more amenable to correction if the underlying strategy causing it can be identified and modified [18]. Thus, we first hypothesized that the feedback training would primarily reduce bias in ensemble perception. As error is a composite of both systematic bias and non-systematic noise, we further hypothesized that any reduction in error would be primarily driven by the correction of this systematic component.

We also hypothesized that the efficacy of the training would be moderated by individual levels of social anxiety for two plausible reasons aligned with prior literature and our training design. One possibility is that individuals with high social anxiety possess a larger initial perceptual bias, and the training would be more effective simply because there is more room for improvement. Alternatively, drawing on findings that anxious individuals show amplified error-related neural responses [19,20], we expected that the high social anxiety group would be more sensitive and responsive to the corrective informational feedback, leading to greater reductions in bias regardless of their baseline levels. Moderation was examined using (a) a continuous trait measure and (b) an exploratory subgroup comparison based on a validated cutoff, to probe a potential non‑linear pattern.

Furthermore, we explored whether the training effect extended to state social anxiety. This exploration is based on the assumption that individuals who are less likely to misinterpret crowds as threatening may feel more confident in social situations.

In sum, this study introduces feedback training as a potential method for correcting ensemble perception biases in social anxiety. By examining both overall effects and moderation by social anxiety, alongside an exploratory state social anxiety hypothesis, this study aims to provide empirical evidence for a targeted intervention that recalibrates ensemble perception relevant to social functioning.

## Materials and methods

### Participants

Adults aged 18 years or older were eligible to participate. Participants were recruited through voluntary sign-ups following announcements made in psychology courses at a university in South Korea. Recruitment was voluntary and open to all students in those courses (i.e., convenience sampling), and both undergraduate and graduate students were included. A total of 121 students participated in the study. One foreign student, who was not fluent in Korean, was excluded from data analysis because all instructions and questionnaires were administered in Korean. No other exclusion criteria were applied. This left a final sample of 120 participants (91 females, 29 males), all of whom were ethnic Koreans. Their ages ranged from 18 to 32 years, with a mean age of 21.53 years (*SD* = 2.35). Participants were randomly assigned to either the training condition (*n* = 60, 47 females) or the control condition (*n* = 60, 44 females).

### Measures

#### Trait social anxiety.

A composite score for trait social anxiety was created by summing all 40 items from the Social Phobia Scale (SPS; [21]) and the Social Interaction Anxiety Scale (SIAS; [21]). The SPS and SIAS are reliable scales that assess different aspects of social anxiety and can be combined to form an overall score [22]. The SPS is a 20-item self-report measure designed to assess anxiety related to performing tasks under potential scrutiny (e.g., speaking or writing in public). The SIAS is a 20-item self-report measure designed to evaluate anxiety experienced during social interactions. Participants rate each statement on a 5-point Likert scale, ranging from 0 (*not at all*) to 4 (*extremely*). We used the Korean versions of the SPS and SIAS [23], and both scales demonstrated excellent internal consistency in this study (Cronbach’s α = .94).

#### State social anxiety.

To assess state social anxiety levels before and after the experimental task, we used a three-item scale from Kashdan et al. [24], originally derived from the Fear of Negative Evaluation Scale [25]. The three items are: “I am afraid that others do not approve of me,” “I am worried that I will say or do the wrong things,” and “I am worried about what other people think of me.” Participants indicated how much they were experiencing these symptoms “now” using a 7-point Likert scale ranging from 1 (*not at all*) to 7 (*extremely*). The internal consistency for the scale in this study was good (Cronbach’s α = .81).

#### Depressive symptoms.

Given that socially anxious individuals often exhibit high levels of depression [26], the Center for Epidemiologic Studies Depression Scale (CES-D; [27]) was administered to control for potential confounding effects. The CES-D is a 20-item self-report measure assessing cognitive, emotional, and physical symptoms of depression. Participants report the frequency of symptoms experienced during the past week on a 4-point Likert scale ranging from 0 (*rarely*) to 3 (*mostly*). We used the Korean version of the CES-D [28], which demonstrated excellent internal consistency in this study (Cronbach’s α = .92).

### Ensemble perception task

#### Stimulus.

**Fig 1:**
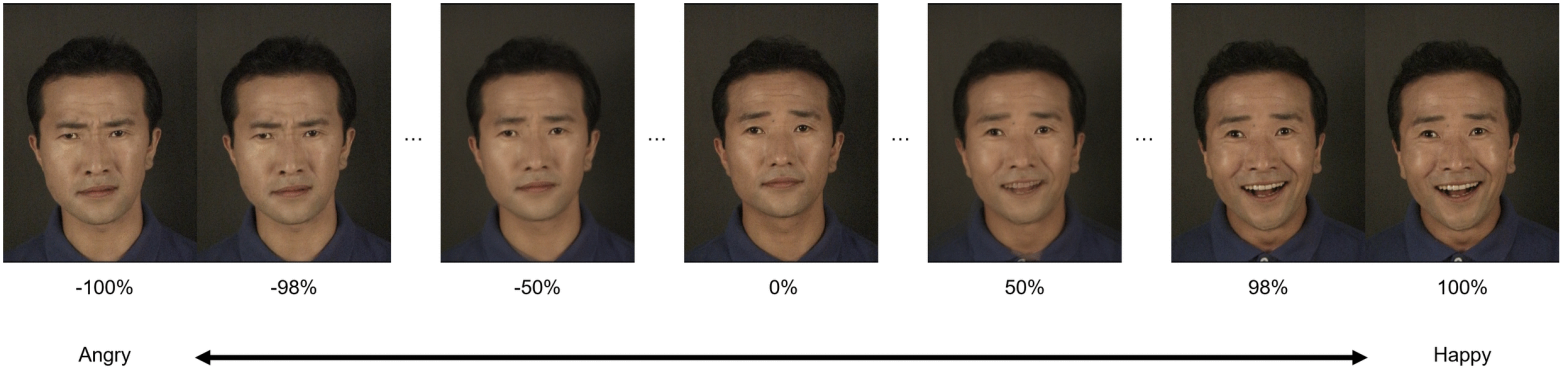
An example of morphed facial stimuli. The morph sequence included equally spaced faces with 2% increments for each model.

Facial stimuli were obtained from a well-validated Korean facial emotion database [29]. A total of seven models were chosen: one for practice, two for the baseline and post-training tests, and four for training. Three prototypical pictures expressing angry, happy, and neutral emotions from each model were selected. A morph sequence was created to gradually change emotional intensity from −100% (extremely angry) to 0% (neutral), and from 0% to 100% (extremely happy). The sequence included equally spaced faces with 2% increments for each model, resulting in 101 intensity levels (i.e., −100, −98,..., 0,..., 98, 100) (Fig 1).

**Fig 2:**
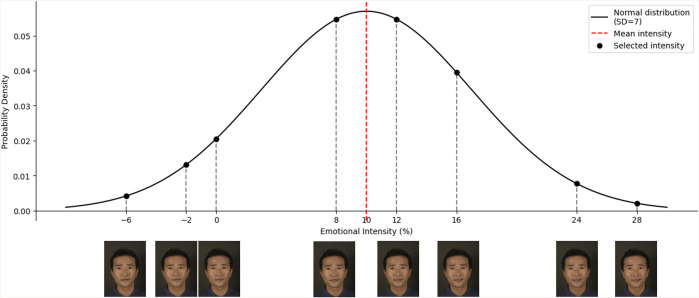
An example of randomly selected intensities from a normal distribution centered at the mean. For convenience, only a limited range of intensity levels is displayed on the x-axis, but the actual minimum and maximum levels were −100% and 100%, respectively.

For the ensemble perception task, the stimulus of each trial was an ensemble of eight morphed facial images. To create seven mean intensity levels (−60, −30, −10, 0, 10, 30, 60), we did not preselect specific intensities. Instead, we randomly sampled eight individual intensities from a normal distribution centered at each mean (*SD* = 7) [30]. This approach increases external validity by presenting a broader range of intensities. To avoid bias in the sampling process, we ensured that four intensity levels were randomly chosen from each side of the mean. For example, if the mean intensity is 10%, the sampled intensities might be −6%, −2%, 0%, 8%, 12%, 16%, 24%, and 28% (Fig 2). Morphing was performed using Abrosoft FantaMorph5 software.

#### Task design.

The task consisted of four blocks: practice, baseline, training, and post-training. Each block used facial ensembles from different models: one female model for practice; one female and one male model for the baseline and post-training blocks; and two female and two male models for the training block.

The trial structure in the practice, baseline, and post-training blocks was identical across both training and control conditions. Each trial began with a fixation cross presented for a random duration between 100 and 500 ms. Following the fixation, a facial ensemble of eight morphed faces was displayed for 250 ms. After the ensemble disappeared, participants rated the mean emotional intensity of the stimulus on a visual analog scale (VAS) ranging from −100% (labeled “extremely angry”) to +100% (labeled “extremely happy”), with an unlabeled tick at the center. Participants moved a marker on the scale using a computer mouse.

**Fig 3:**
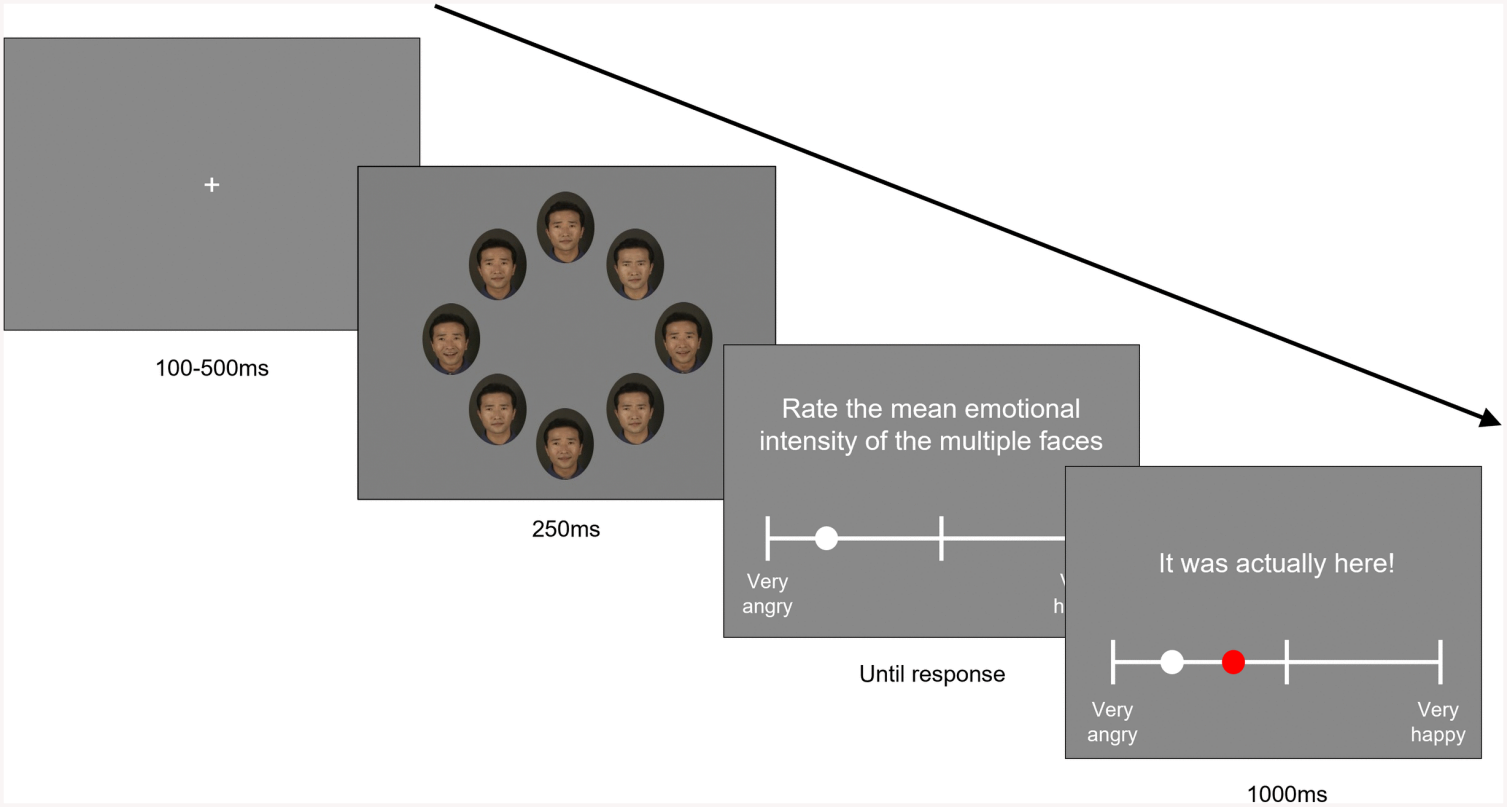
Example of a trial from the training block in the ensemble perception task. The final feedback screen displaying both the participant’s rating and the actual mean intensity was presented only in the training condition.

The training block differed by condition. In the training condition, after each rating, feedback was provided for 1000 ms, displaying the actual mean intensity of the stimulus alongside the participant’s rating on the scale (Fig 3). In contrast, the control group received no feedback.

The practice block consisted of seven trials (one for each of the seven mean intensity levels). The baseline block comprised 70 trials (10 repetitions for each mean intensity). The training block consisted of 280 trials (40 repetitions for each mean intensity), with an optional break at the halfway point. The post-training block was identical to the baseline block (70 trials). In all blocks, the order of trials was randomized for each participant. The task was created using PsychoPy 2021.2.0 [31] and presented on a 15.6-inch laptop computer.

### Procedure

The study was approved by the Institutional Review Board of The Catholic University of Korea (No. 1040395-202410-02). Participants were recruited between November 4 and December 10, 2024. After providing written informed consent, participants were randomly assigned to either the training or the control condition. The study was conducted in a laboratory at the university. Upon arrival, participants were escorted to an individual cubicle. First, they completed a set of baseline questionnaires (SPS, SIAS, state social anxiety scale, CES-D) via Google Forms. Next, they began the ensemble perception task. An experimenter supervised the practice block to ensure comprehension. After the practice block, the experimenter left the room, and participants completed the remaining blocks alone in a dark room. The task took approximately 15–20 minutes. Immediately following the task, participants completed the state social anxiety scale again. Participants were then debriefed and compensated with 2,000 KRW (approximately 1.50 USD). The entire procedure lasted about 25–30 minutes.

### Data analysis

#### Data preparation.

Two primary outcome variables were derived from the task. Ensemble perception bias was calculated by subtracting the actual mean emotional intensity of the facial crowd from the perceived intensity (i.e., the participant’s rating). A negative value indicates a more negative perception than the actual mean, and a positive value indicates a more positive perception. Ensemble perception error was calculated as the absolute value of the difference between perceived and actual intensity (|perceived − actual|). For simplicity, we henceforth refer to these variables as bias and error, respectively.

#### Statistical analyses.

All statistical analyses were conducted using IBM SPSS Statistics for Windows, version 28.0 [32]. First, descriptive statistics and Pearson correlations for baseline variables were calculated. Second, to ensure group equivalence, a chi-square test was used for gender distribution, and independent samples *t*-tests were conducted for demographic and baseline variables.

To test the primary hypotheses, a series of one-way analyses of covariance (ANCOVAs) were conducted. The experimental condition (training vs. control) served as the independent variable, and post-training bias, error, and state social anxiety scores were entered as dependent variables in separate models. The corresponding baseline score for each dependent variable was included as a covariate. This approach provides a more statistically robust test of post-training group differences than a repeated-measures analysis of variance (ANOVA).

To test the moderating effect of trait social anxiety, a moderation analysis was performed using the PROCESS macro for SPSS (Model 1; [33]). In this model, the experimental condition was the independent variable, the post-training outcome scores were the dependent variables, and trait social anxiety was the moderator, with the baseline score of each outcome variable included as a covariate.

Furthermore, an exploratory subgroup analysis was conducted. Participants were divided into high and low social anxiety groups based on a cutoff score of >66 on the composite score for trait social anxiety (i.e., the sum of the SPS and SIAS). This cutoff has been validated as the optimal threshold for identifying individuals with social anxiety disorder in a Korean population [34]. Based on this criterion, 35 participants (30 females) were classified into the high social anxiety group and 85 participants (61 females) into the low social anxiety group. The gender distribution between the two groups did not significantly differ, χ²(1, *N* = 120) = 2.63, *p* = .105. For each subgroup, separate ANCOVAs were run with the experimental condition as the independent variable and post-training scores as dependent variables, controlling for respective baseline scores. Levene’s test was used to check the assumption of homogeneity of variances. When this assumption was violated, parameter estimates with robust standard errors (HC3 method) were reported. Finally, all analyses were repeated with baseline depressive symptoms included as an additional covariate to ensure the robustness of the findings.

## Results

### Preliminary analyses

Descriptive statistics and Pearson correlation coefficients for the baseline variables are presented in Table 1. At baseline, bias was significantly and negatively correlated with error (*r* = −.56, *p* < .001), trait social anxiety (*r* = −.24, *p* = .009), and depressive symptoms (*r* = −.30, *p* < .001). Trait social anxiety was strongly and positively correlated with baseline state social anxiety (*r* = .69, *p* < .001) and depressive symptoms (*r* = .58, *p* < .001).

**Table 1:** Descriptive statistics and Pearson correlation coefficients for baseline variables.

Variable	1	2	3	4	Mean	*SD*
1. Baseline ensemble perception bias	—				−7.06	8.04
2. Baseline ensemble perception error	−.56^***^	—			26.11	5.09
3. Baseline state social anxiety	−.18	.02	—		11.53	4.31
4. Trait social anxiety	−.24^**^	.11	.69^***^	—	54.29	28.93
5. Depressive symptoms	−.30^***^	.09	.49^***^	.58^***^	14.54	9.46

*Note. N* = 120. ^**^ *p* < .01, ^***^ *p* < .001.

Independent samples *t*-tests confirmed the equivalence of the training and control groups on demographic and baseline variables. As shown in Table 2, there were no statistically significant differences between the groups in age or any baseline variable (all *p* > .05). A chi-square test also indicated no significant difference in gender distribution between the groups, χ²(1, *N* = 120) = 0.41, *p* = .522, confirming that random assignment was successful.

**Table 2:** Group equivalence on demographic and baseline variables.

Variable	Training	Control	*t*	*p*	95% CI	Cohen’s *d*
	Mean (*SD*)	Mean (*SD*)				
Age	21.65 (2.39)	21.40 (2.32)	0.58	.562	[-0.60, 1.10]	0.11
Baseline ensemble perception bias	−7.21 (8.47)	−6.90 (7.66)	−0.21	.833	[-3.23, 2.61]	−0.04
Baseline ensemble perception error	26.12 (5.85)	26.10 (4.24)	0.03	.977	[-1.82, 1.87]	0.01
Baseline state social anxiety	11.05 (4.48)	12.02 (4.12)	−1.23	.221	[-2.52, 0.59]	−0.23
Trait social anxiety	52.98 (31.29)	55.60 (26.57)	−0.49	.622	[-13.11, 7.88]	−0.09
Depressive symptoms	13.03 (9.16)	16.05 (9.58)	−1.76	.081	[-6.41, 0.37]	−0.32

*Note.* CI = Confidence interval.

### Overall training effect

A series of ANCOVAs were conducted on the post-training outcomes, controlling for their respective baseline scores. The feedback training demonstrated a significant effect on post-training bias (*F*(1, 117) = 3.92, *p* = .050, partial η² = .032) and a marginally significant effect on post-training error (*F*(1, 117) = 3.80, *p* = .054, partial η² = .031). However, the training did not have a significant effect on post-training state social anxiety (*F*(1, 117) = 0.14, *p* = .712, partial η² = .001).

### Training effect moderated by trait social anxiety

A moderation analysis using the PROCESS macro revealed that the interaction between training condition and trait social anxiety was not statistically significant for post-training bias (*b* = 0.02, *t* = 0.49, *p* = .623, 95% CI [−0.07, 0.11]), post-training error (*b* = −0.02, *t* = −0.61, *p* = .540, 95% CI [−0.08, 0.04]), or post-training state social anxiety (*b* = −0.02, *t* = −0.84, *p* = .404, 95% CI [−0.06, 0.03]).

### Exploratory subgroup analyses

Although the formal moderation analysis was not significant, exploratory ANCOVAs were conducted separately for the high and low social anxiety subgroups based on a validated cutoff for trait social anxiety. Prior to training, the two groups did not differ in baseline bias, *t*(48.56) = 1.18, *p* = .245. Similarly, baseline error scores were not significantly different between the groups, *t*(118) = −0.13, *p* = .895.

Nevertheless, a significant post-training improvement was observed only in the high social anxiety group. For the high social anxiety group, the training had a significant effect on reducing post-training ensemble perception bias (*F*(1, 32) = 5.21, *p* = .029, partial η² = .140). The analysis for post-training ensemble perception error revealed a violation of the homogeneity of variances assumption (*p* = .020). An analysis with robust standard errors confirmed a significant effect of the training (*t* = 2.08, *p* = .046, 95% CI [0.07, 7.37]).

In contrast, for the low social anxiety group, the effect of the training was not statistically significant for bias (*F*(1, 82) = 1.15, *p* = .287, partial η² = .014) or error (*F*(1, 82) = 0.72, *p* = .398, partial η² = .009). The training’s effect on state social anxiety was not significant in either the high (*F*(1, 32) = 2.19, *p* = .149, partial η² = .064) or low (*F*(1, 82) = 0.16, *p* = .692, partial η² = .002) social anxiety group.

**Fig 4:**
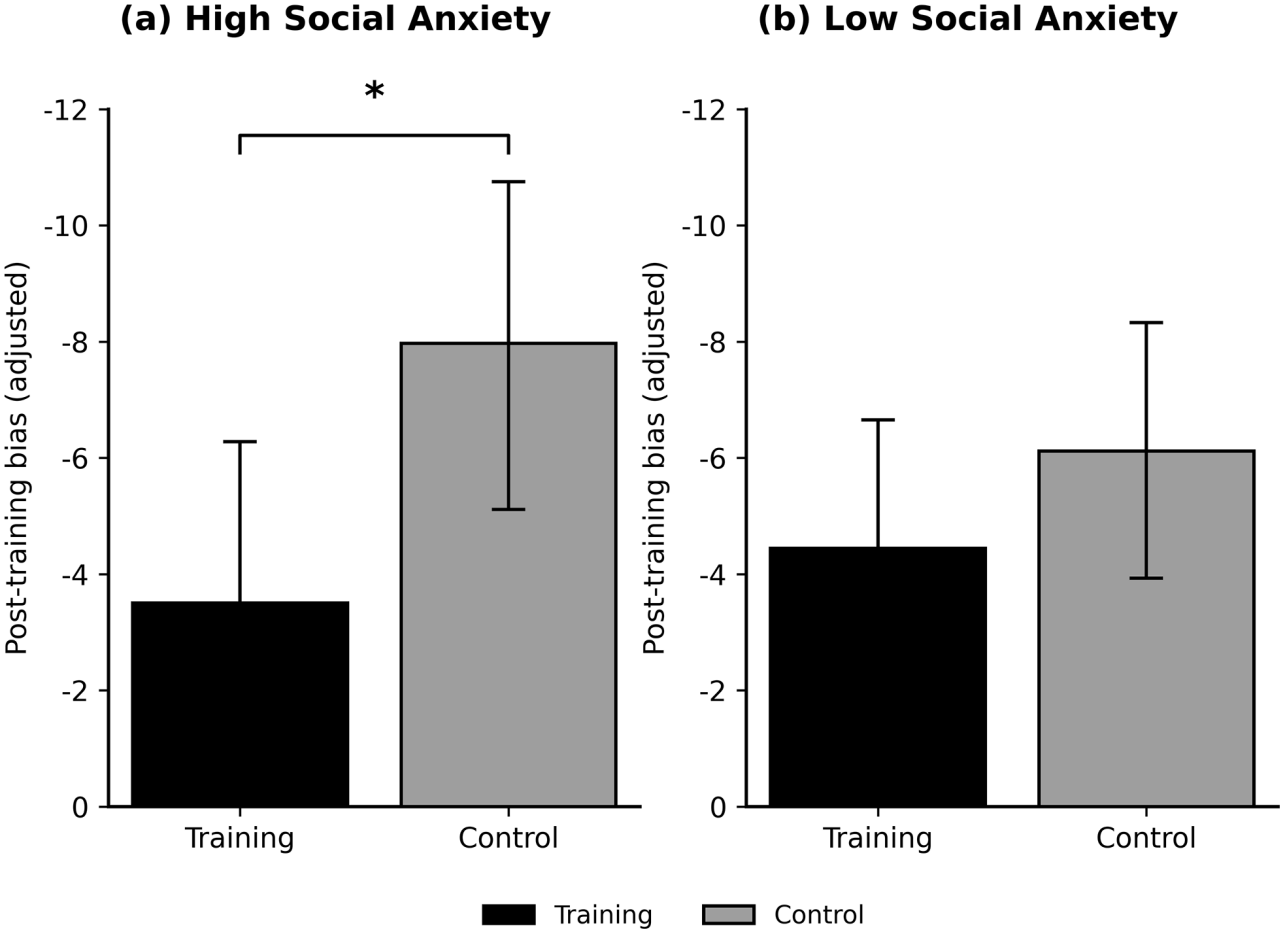
Adjusted post-training ensemble perception bias by condition and social anxiety group. Panel (a) depicts results for the High social anxiety group, and panel (b) for the Low social anxiety group. Bars represent adjusted post-training ensemble perception bias derived from ANCOVA analyses, with Training (black) and Control (gray) conditions. Negative values correspond to stronger negative perceptual bias. Error bars indicate 95% confidence intervals around adjusted means. ^*^ *p* < .05.

Fig 4 illustrates the adjusted post-training ensemble perception bias by condition (training vs. control) and social anxiety group.

### Additional analyses: Controlling for depressive symptoms

To ensure that the observed effects were not confounded by depressive symptoms, all primary analyses were repeated with baseline CES-D scores included as a covariate.

After controlling for baseline depressive symptoms, the main effect of the training condition on post-training ensemble perception bias (*F*(1, 116) = 3.09, *p* = .082, partial η² = .026), ensemble perception error (*F*(1, 116) = 2.95, *p* = .089, partial η² = .025), and state social anxiety (*F*(1, 116) = 0.07, *p* = .793, partial η² = .001) were not significant.

The moderation analysis revealed no significant interaction effect between the training condition and trait social anxiety for post-training ensemble perception bias (*b* = 0.02, *t* = 0.43, *p* = .667, 95% CI [−.07,.11]), post-training ensemble perception error (*b* = −0.02, *t* = −0.59, *p* = .558, 95% CI [−.08,.05]), or post-training state social anxiety (*b* = −0.02, *t* = −0.83, *p* = .411, 95% CI [−.06,.03]).

The exploratory analysis on subgroups showed a similar but weaker pattern. For the high trait social anxiety group, the training effect remained significant for post-training ensemble perception bias (*F*(1, 31) = 5.35, *p* = .028, partial η² = .147). However, the effect of the training was no longer statistically significant for post-training ensemble perception error (*t* = 1.97, *p* = .058, 95% CI [−0.12, 7.18]). For the low trait social anxiety group, the effects remained non-significant for post-training ensemble perception bias (*F*(1, 81) = 0.41, *p* = .523, partial η² = .005) and post-training ensemble perception error (*F*(1, 81) = 0.42, *p* = .521, partial η² = .005). Similarly, the effect on post-training state social anxiety was not significant in the high (*F*(1, 31) = 2.19, *p* = .149, partial η² = .066) or low (*F*(1, 81) = 0.37, *p* = .543, partial η² = .005) social anxiety group.

## Discussion

This study examined whether feedback training could reduce bias and error in the ensemble perception of facial crowds and whether these effects varied by social anxiety level. The findings suggest that feedback training primarily reduced perceptual bias, with more pronounced effects among individuals with higher trait social anxiety.

Before training, baseline analyses showed that error was not significantly related to trait social anxiety, whereas bias was. Specifically, individuals with high social anxiety tended to perceive facial crowds as more negative, indicating a greater negative bias in ensemble perception. Because error reflects the combined influence of both systematic bias and non-systematic noise [18], this selective relationship suggests that social anxiety is particularly associated with the systematic component of perceptual distortion. This supports the rationale that interventions for social anxiety should target negative perceptual bias.

At the overall level, the training significantly reduced bias but had a limited effect on error. This pattern aligns with theoretical distinctions between bias and noise. Bias represents a directional, systematic deviation that can be corrected through strategy adjustment, whereas noise reflects random fluctuations that are less amenable to short-term change [18]. Thus, interventions that deliver explicit feedback on the magnitude and direction of deviation may effectively recalibrate bias, while leaving random noise largely unaffected.

When treating social anxiety as a continuous variable, moderation analyses revealed no significant interaction. However, exploratory subgroup analyses based on a validated cutoff indicated a selective improvement among participants with high social anxiety. The training significantly reduced both bias and error in this group, whereas no meaningful change was observed in the low social anxiety group.

Notably, the mean difference in baseline bias between the high and low social anxiety groups did not reach statistical significance. This discrepancy suggests that the key to the training’s selective efficacy lies not in the initial magnitude of the bias itself, but rather in the underlying psychological mechanisms that govern how individuals with high social anxiety respond to corrective feedback.

Individuals with high social anxiety may possess heightened error-monitoring sensitivity, which could amplify their responsiveness to corrective feedback. Previous evidence shows that anxious individuals exhibit increased neural sensitivity to their own errors, reflected in amplified error-related negativity (ERN) signals [19,20]. While EEG was not recorded, high social anxiety participants may have perceived the discrepancy between their judgment and the objective feedback—the “error gap”—as a more salient and motivating signal, enhancing engagement and facilitating faster strategy adjustment. Crucially, the feedback was informational rather than evaluative; participants were shown only their rating alongside the objective mean intensity, without evaluative cues such as “correct/incorrect.” This design likely leveraged intrinsic error sensitivity for constructive recalibration while minimizing evaluative threat. The absence of any increase in state social anxiety supports this interpretation, suggesting the procedure enhanced accuracy without eliciting threat-related arousal.

This interpretation is strongly substantiated by the fundamental nature of the errors across the two groups. In the high social anxiety group, we observed a robust negative correlation between baseline bias and baseline error (*r* = −.82), a relationship significantly stronger than that of the low social anxiety group (*r* = −.38; *z* = 3.59, *p* < .001). This structural difference suggests that for high social anxiety individuals, ‘error’ was not random noise but a direct manifestation of their systematic negative bias. Consequently, their heightened sensitivity to error effectively might function as a sensitivity to their own bias, providing a powerful intrinsic motivation for correction. For the low social anxiety group, whose errors were predominantly non-systematic, the feedback provided no consistent corrective signal.

This feedback-driven correction, motivated by heightened sensitivity to biased errors, likely operates by modifying the specific cognitive strategies known to generate the bias. One such mechanism is the recalibration of attentional control [35,36]. Socially anxious individuals are known to direct attention more rapidly to negative stimuli and struggle to disengage from them [37,38] while allocating less attention to positive expressions [39]. Within a facial crowd, this may manifest as an over-weighting of the most negative faces when computing the average emotion, as attention to a specific face increases its weight in mean extraction [40]. Feedback may have helped participants shift toward a more balanced and distributed attentional strategy. A second, complementary mechanism involves the correction of interpretation bias. Social anxiety is characterized by a tendency to interpret ambiguous stimuli as threatening [41]. When confronted with neutral or mildly expressive faces, socially anxious individuals may default to a more negative interpretation. Feedback training can correct this bias for individual faces [16], and our results suggest this may extend to facial crowds.

Despite these promising changes in perception, the single-session training did not significantly reduce state social anxiety. This is unsurprising, as a single-session change in a low-level perceptual task may be insufficient to alter a higher-order emotional state without opportunities to apply this change in real-world social contexts.

To account for comorbid depression, depressive symptoms were included as a covariate. Although depressive symptoms were related to the outcomes, the key effect of bias reduction in the high social anxiety group remained robust. This suggests that the training specifically targets social anxiety–related cognitive processing rather than general negative affectivity.

Clinically, these results suggest that individualized, non-evaluative corrective feedback can effectively engage the cognitive mechanisms underlying social anxiety. Even within a single session, participants learned to recognize and correct their consistent negative averaging tendencies. This highlights the potential of brief, structured, feedback-based interventions that could be implemented digitally or as early preventive programs for subclinical social anxiety.

Several limitations should be noted. First, feedback was based on the objective mean intensity of facial crowds (i.e., the mathematical average of the morph values), which may not perfectly correspond to each individual’s subjective perceptual mean [42]. This discrepancy means that the feedback, while objectively correct, may have been perceptually inaccurate for some participants. In this case, feedback could have been misleading, potentially confounding the results. Second, using morphed facial expressions from a single model limits ecological validity. Third, only immediate post-training effects of a single session were assessed. Finally, because participants were Korean university students recruited through a convenience sampling method, the generalizability of the findings is limited.

## Conclusion and recommendation

In this study, feedback training refers to a learning process in which participants received trial-by-trial information comparing their perceptual judgments with the objective mean of facial crowd emotions. This individualized and non-evaluative feedback was designed to facilitate the recalibration of biased ensemble perception (i.e., systematic deviations in the perceived average emotion of facial crowds).

The present study provides evidence that feedback training is a promising intervention for modifying systematic perceptual biases in individuals with high social anxiety. Based on the findings and limitations, we recommend several directions for future research. The selective effect in the high social anxiety group points toward a potential non-linear relationship between social anxiety and feedback sensitivity. However, this possibility should be considered exploratory because it was not directly tested here and should be examined in future pre-registered research.

To elucidate the specific cognitive mechanisms underlying the training effect, future studies could examine changes in attentional allocation using eye-tracking or include a dual-task design involving both ensemble and single-stimulus perception. Additionally, future research should address the limitation of using an objective mean for feedback. Rather than relying on objective morph values, we recommend that future studies first establish a perceptually calibrated mean based on pilot data from a representative sample. This would ensure that the feedback provided during training aligns more closely with participants’ subjective experiences, thereby enhancing both the accuracy and validity of the intervention.

To improve ecological validity, future research should employ multi-identity crowds with diverse emotional intensities. To evaluate the durability of bias correction and its clinical relevance, studies should implement multi-session training paradigms with long-term follow-up phases, assessing whether perceptual changes translate into reductions in social anxiety symptoms. Future research should also incorporate diagnostic interviews for social anxiety disorder and recruit participants across broader age ranges and cultural backgrounds.

Ultimately, reducing negative perception of group emotions may help socially anxious individuals feel more confident and less threatened in everyday social contexts.
